# Longitudinal trajectories of physical fitness among university students: evidence from three-year linked annual fitness records for campus health promotion

**DOI:** 10.3389/fpubh.2026.1921184

**Published:** 2026-09-24

**Authors:** Dongkai Li, Mingyang Wang, Yunchen Meng, Xuecheng Zheng, Ritai Chen, Hao Kong, Sanjun Yang

**Affiliations:** 1 Department of Physical Education and Research, China University of Mining and Technology, Beijing, China; 2 School of Sports Training, Tianjin University of Sport, Tianjin, China

**Keywords:** discipline group, health promotion, longitudinal study, mixed-effects model, physical fitness, university students

## Abstract

**Background:**

University is a critical period for the transition from adolescence to early adulthood, during which lifestyle and physical activity patterns become more stable. This study examines longitudinal changes in physical fitness among university students and evaluates whether trajectories differ by discipline group, baseline grade, and sex.

**Methods:**

Annual physical fitness test records from 2023 to 2025 are linked at the student level. A total of 4,406 students contribute 12,795 student-year observations. Multilevel linear mixed-effects models are used to analyze total physical fitness score, with grade-based outcomes and raw item measurements used as complementary analyses.

**Results:**

Total physical fitness score declines significantly from 2023 to 2025 in all discipline groups. The year × discipline-group interaction is significant, indicating different longitudinal trajectories. The largest adjusted decline is observed in Science, followed by HSSM and Engineering. Pass rates decline in all groups, while the loss of good-or-excellent status is more pronounced in Science and HSSM. Raw measurements suggest that the decline is concentrated mainly in speed, endurance, lower-limb explosive power, and muscular strength or endurance.

**Conclusion:**

University students show a marked decline in physical fitness over 3 years. These findings support the use of linked annual fitness records as a campus health surveillance tool to identify declining fitness trajectories and guide stage-specific, risk-stratified health promotion strategies.

## 1 Introduction

University is an important transition from late adolescence to early adulthood. During this period, physical maturation is largely complete, while lifestyle, physical activity habits, sedentary behavior, and weight status gradually become more stable. Physical fitness at this stage is therefore not only related to current physical function and exercise capacity, but also provides a foundation for adapting to academic life and maintaining healthy behavior. Recent evidence shows that insufficient physical activity and sedentary behavior are common among young people (1–5), and declining physical fitness among university students has become an increasing concern for campus health management (6–10). If this decline continues during university, it may have adverse implications for later cardiometabolic health and chronic disease risk (11).

This relevance is further supported by recent evidence showing that physical fitness, especially cardiorespiratory fitness, is increasingly recognized as a key marker of long-term cardiovascular and general health (12).

However, much of the available evidence on university students’ physical fitness is based on cross-sectional comparisons, repeated population-level surveys, or analyses focused on single fitness indicators (6–10). These designs are valuable for describing population trends, but they cannot fully characterize within-student trajectories. Evidence is also limited regarding whether longitudinal changes in overall fitness differ by academic discipline, stage of university study, or sex, and which specific fitness domains account for those changes. Individually linked annual physical fitness records can therefore provide complementary evidence by distinguishing population-level trends from within-student change over time.

To examine how physical fitness changes during university, this study follows students who are freshmen or sophomores in 2023 for three consecutive years. As university life progresses, course arrangements, academic tasks, participation in physical education, and time available for self-directed exercise may all change, and these factors may jointly shape physical fitness trajectories (4, 5, 13–15). Academic disciplines also correspond to different training plans and learning environments. Engineering, Science, and humanities, social sciences, and management students may differ in curriculum structure, practical teaching, and time allocation. In this study, discipline group is treated as a background indicator of the educational environment, with the aim of examining whether longitudinal physical fitness trajectories differ across these groups.

On this basis, this study has three objectives: to estimate the longitudinal change in total physical fitness score among university students, to test whether this change differs by discipline group, baseline grade, and sex, and to identify which grade-based outcomes and raw fitness domains are most involved in the observed decline. The study is guided by three working hypotheses: total physical fitness score declines during university; the magnitude of decline differs across discipline groups, baseline grade, and sex; and the decline is concentrated mainly in fitness domains that depend on sustained aerobic activity, strength-related training, and sufficient exercise intensity. The analysis applies a multilevel linear mixed-effects model to physical fitness records from 2023 to 2025, retaining individual differences in baseline fitness and rates of change while accounting for clustering at the major level and repeated measurements from the same student.

## 2 Materials and methods

### 2.1 Participants

Participants are drawn from the university’s annual physical fitness test records for 2023, 2024, and 2025. The baseline year is 2023. Students are included if they are freshmen or sophomores at baseline and have an identifiable student ID, total physical fitness score, sex, major, and discipline-group information. The final sample includes 4,406 students, of whom 4,071 complete all three annual tests, 247 have two annual records, and 88 have one annual record. These records form 12,795 student-year observations. The primary analysis retains all available records from eligible students to reduce the selection bias that may arise from restricting the analysis to complete 3-year follow-up cases. Each observation represents one student’s physical fitness test result in a given year. Student ID is used to link repeated measurements across years and to account for within-student correlation; baseline major is used to identify clustering at the major level. All eligible students in 2023 are included without additional sampling or *a priori* sample-size selection based on a target effect size. Estimation precision is expressed through effect estimates and 95% confidence intervals.

### 2.2 Discipline grouping and covariates

Students are classified into three discipline groups according to their baseline major in 2023: Engineering, Science, and humanities, social sciences, and management (HSSM). The classification covers 33 majors. The Engineering group mainly includes students from schools such as Mechanics and Civil Engineering, Chemical and Environmental Engineering, Mechanical and Electrical Engineering, Geoscience and Surveying Engineering, Emergency Management and Safety Engineering, Energy and Mining Engineering, and Artificial Intelligence, with majors including safety engineering, civil engineering, chemical engineering and technology, mechanical engineering, geological engineering, electrical engineering and automation, computer science and technology, environmental engineering, mineral processing engineering, surveying and mapping engineering, artificial intelligence, and mining engineering. The Science group includes mathematics and applied mathematics and geophysics. The HSSM group includes majors such as business administration, public administration, accounting, big data management and application, English, industrial engineering, and law. The discipline group is determined by the baseline major and remains fixed throughout follow-up.

The sex distribution differs clearly across the three discipline groups at baseline. The Engineering group includes 3,284 students, with 2,542 males (77.4%) and 742 females. The Science group includes 342 students, with 244 males (71.3%) and 98 females. The HSSM group includes 780 students, with 466 females (59.7%) and 314 males. The baseline grade distribution is similar across groups: the Engineering group includes 1,656 freshmen and 1,628 sophomores; the Science group includes 177 freshmen and 165 sophomores; and the HSSM group includes 389 freshmen and 391 sophomores. Because sex and baseline grade may both relate to total physical fitness score and its subsequent change, both variables are included in the model and their interactions with year are examined. This discipline grouping is used because different academic fields may vary in training plans, course organization, and learning tasks. Engineering majors usually include laboratory work, engineering training, internships, and practical teaching. Science majors emphasize basic theory, laboratory analysis, and sustained academic study. HSSM majors involve more reading, writing, case analysis, language expression, and social science training. These educational features may correspond to different learning rhythms, time allocation, and opportunities for campus activity.

In this study, discipline group is used as a background variable that reflects differences in professional training environments and supports comparisons of longitudinal trajectories in total physical fitness score. To reduce the influence of baseline differences among specific majors on discipline-group comparisons, major is further included as a random effect. Because the Science group consists of mathematics and applied mathematics and geophysics in this sample, its estimates should be interpreted in relation to the science majors represented in the present university context.

### 2.3 Physical fitness assessment

The physical fitness test for Chinese university students is an important component of student health monitoring and physical education evaluation (16, 17). Through annual standardized testing, it tracks body morphology, physical function, and motor fitness, and provides evidence for physical education, health promotion, and student fitness intervention. The test is usually organized by academic year, and universities collect data, assign scores, and classify fitness grades according to unified test items and scoring standards. Compared with evaluation based on a single sport-specific performance, the university physical fitness test emphasizes an integrated assessment of students’ overall physical health.

The assessment is implemented according to the National Student Physical Fitness Standard rather than being selected from the Eurofit battery. Some domains, such as flexibility, speed, jumping ability, endurance, and muscular fitness, overlap conceptually with international youth fitness batteries, but the test items, scoring rules, and grade thresholds in this study follow the Chinese university testing system.

The test items used in this study cover several dimensions of physical fitness. BMI is mainly used to assess body morphology and weight status. Vital capacity reflects lung function and cardiopulmonary-related capacity (18, 19). The 50-m sprint assesses speed and short-distance explosive ability (20, 21). The standing long jump reflects lower-limb explosive power and body coordination (22, 23). Sit-and-reach assesses flexibility. Sex-specific items include the 800-m run for females and the 1,000-m run for males, which assess aerobic and cardiorespiratory endurance; the one-minute sit-up test for females, which reflects abdominal strength and muscular endurance; and pull-ups for males, which reflect upper-body strength and muscular endurance. Together, these items form a multidimensional assessment system for university students’ physical fitness.

During testing, each item is administered by trained testing staff and physical education administrators according to standardized procedures. Results are first recorded as raw measurements and then converted into item scores according to the corresponding scoring criteria. The total physical fitness score is calculated from item scores and further classified into four grades: fail, pass, good, and excellent. The test month, venue, equipment, scoring standards, and organization procedures remain consistent across the 2023, 2024, and 2025 assessments, which supports comparability across years.

### 2.4 Outcomes

Annual physical fitness testing is conducted according to the National Student Physical Fitness Standard (16). Test outputs include raw item measurements, item scores, total physical fitness score, and fitness grade. Grades are defined according to the academic-year total score: excellent for 90.0 points or above, good for 80.0–89.9 points, pass for 60.0–79.9 points, and fail for 59.9 points or below. The total physical fitness score is the primary outcome and is used to evaluate longitudinal changes in overall physical fitness. Passing, good or excellent, and excellent grades are analyzed as supplementary outcomes to determine whether changes in total score translate into changes in fitness-grade status. Standard scores are obtained by converting raw item measurements into item scores according to sex-specific standards and then weighting each item according to the official scoring scheme. BMI accounts for 15%, vital capacity for 15%, the 50-m sprint for 20%, sit-and-reach for 10%, standing long jump for 10%, strength for 10%, and endurance running for 20%. The strength item is pull-ups for males and one-minute sit-ups for females; the endurance item is the 1,000-m run for males and the 800-m run for females. Additional points may be awarded for performance above the full-score standard in pull-ups, the 1,000-m run, one-minute sit-ups, and the 800-m run. Each type of bonus item can add up to 10 points, with a maximum total bonus of 20 points. Therefore, the academic-year total score consists of weighted standard scores and corresponding bonus points. Because the BMI item score is already included in the total physical fitness score, raw BMI is not used as a routine covariate in the primary total-score model. Instead, the BMI item score is removed from the total score in sensitivity analysis to examine whether the main findings depend on the BMI-score component.

The standard-score component can be summarized as follows: standard score = 0.15 × BMI score + 0.15 × vital capacity score + 0.20 × 50-m sprint score + 0.10 × sit-and-reach score + 0.10 × standing long jump score + 0.10 × strength score + 0.20 × endurance run score. Strength corresponds to pull-ups in males and one-minute sit-ups in females; endurance running corresponds to the 1,000-m run in males and the 800-m run in females.

### 2.5 Data processing and quality control

Raw physical fitness data are organized by year and linked across 2023, 2024, and 2025 using student ID, resulting in a long-format student-year dataset. Data cleaning includes consistency checks for student ID, year, sex, baseline grade, major, discipline group, total physical fitness score, and fitness grade. Discipline group is determined by the 2023 baseline major and remains unchanged during follow-up. Before analysis, student IDs are anonymized, and direct identifiers such as names and student numbers are not retained.

For raw item measurements, variable names, measurement units, and recording formats are standardized. The minute-second records for the 800-m and 1,000-m runs are converted into seconds. Records with clear format errors or values outside plausible measurement ranges that cannot be verified are treated as missing only for the corresponding item-level analysis and do not affect the total-score or grade-based analyses.

The primary analysis retains all available records from students who meet baseline inclusion criteria and does not routinely impute missing annual total scores. Under an assumption that missingness is approximately random conditional on observed variables, the linear mixed-effects model uses each student’s available one to three measurements for estimation. To further assess the possible influence of loss to follow-up, inverse probability weighting and missing-outcome stress tests are conducted as sensitivity analyses.

No newly developed questionnaire is used in this study, and no questionnaire validation procedure is therefore applicable. The study also does not introduce a new field-testing protocol requiring a pilot survey; instead, it analyzes routinely collected annual physical fitness monitoring records generated under the university’s standardized testing procedures.

### 2.6 Statistical analysis

#### 2.6.1 Primary mixed-effects analysis

Total physical fitness score is analyzed as a continuous outcome using a linear mixed-effects model. Fixed effects include year, discipline group, baseline grade, and sex, with interaction terms between year and discipline group, year and baseline grade, and year and sex. The model also includes a random intercept for major, a student-specific random intercept, and a student-specific time slope to account for clustering at the major level, individual differences in baseline fitness, and individual rates of change. The model is specified as follows:


$$\begin{array}{c} {\text{Total score}}_{i}j=\beta_{0}+\beta_{1}{\text{Year}}_{\mathrm{ij}}+\beta_{2}{\text{Group}}_{i}+\beta_{3}({\text{Year}}_{\mathrm{ij}}\times {\text{Group}}_{i})\\ +\beta_{4}{\text{Baseline grade}}_{i}+\beta_{5}({\text{Year}}_{i}j\times {\text{Baseline grade}}_{i})\\ +\beta_{6}\mathrm{Se}x_{i}+\beta_{7}(\mathrm{Yea}r_{i}j\times \mathrm{Se}x_{i})+u_{m}+b_{0}i\\ +b_{1}\text{iTim}e_{i}j+\varepsilon_{i}j \end{array}$$

In the model, u_m_ denotes the random intercept for major; *b*₀ᵢ and *b*₁ᵢ denote the student-specific random intercept and random time slope, respectively; and *ε*_ij_ denotes the residual for student *i* in year *j*. Year is entered as a categorical variable, and within-student residuals follow a first-order autoregressive correlation structure, AR(1). After comparing alternative random-effect and residual-correlation structures, the final model uses the random-slope plus AR(1) specification because its parameters are identifiable and the model provides stable fit. The year effect estimates the overall time trend. The year × discipline-group interaction tests whether trajectories differ across the three discipline groups. The year × baseline-grade and year × sex interactions examine whether changes differ by entry grade and sex. Fixed effects are reported as F statistics with degrees of freedom and *p* values. Adjusted means and 95% confidence intervals are calculated for each discipline group by year. Changes from 2023 to 2024, from 2024 to 2025, and from 2023 to 2025 are also estimated to describe the magnitude and timing of decline.

#### 2.6.2 Grade-based and raw-measurement analyses

Passing and good-or-excellent status are analyzed as binary outcomes using conditional logistic regression stratified by student ID. These models include interactions of year with discipline group, baseline grade, and sex, and robust standard errors are calculated with major as the clustering unit. Because conditional logistic regression mainly uses within-student comparisons among students whose grade status changes, the number of students contributing information to each estimate is also reported. To assess the influence of major-level clustering and the limited number of majors, 500 stratified cluster bootstrap samples are drawn by discipline group to obtain bootstrap confidence intervals for odds ratios and to test the year × discipline-group interaction. Excellent status is analyzed descriptively because events are sparse. Raw item measurements are used as exploratory outcomes to help identify the physical domains involved in changes in total score. Sex is adjusted for in models of common test items, while the 800-m run, one-minute sit-ups, 1,000-m run, and pull-ups are analyzed separately within the corresponding sex.

#### 2.6.3 Sensitivity analyses and model diagnostics

Sensitivity analyses include a complete 3-year follow-up analysis, removal of the BMI item score from the total score, testing of the year × discipline group × sex interaction, inverse probability weighting for missing data, missing-outcome stress tests, leave-one-major-out analysis, and outlier handling. In inverse probability weighting, the probability of having a 2025 physical fitness record is estimated using baseline total score, BMI, discipline group, sex, baseline grade, and major, and stabilized weights are calculated. To reduce the influence of extreme weights, weights are truncated at the 1st and 99th percentiles. The missing-outcome stress test is designed as a conservative sensitivity scenario rather than an assumption about the true missing outcomes. It assumes that the 2025 total score of students with missing outcomes is 2–5 points lower than the conditional predicted value, thereby assessing whether the main conclusions remain stable under progressively unfavorable missing-data scenarios. Leave-one-major-out analysis removes one major at a time and refits the primary model to evaluate whether the year × discipline-group interaction is driven by any single major. Outlier sensitivity analyses winsorize total scores at the 0.5th and 99.5th percentiles and refit the model after excluding observations with absolute standardized residuals greater than 4. Model diagnostics include convergence status, identifiability of variance parameters, residual-versus-fitted plots, and normal *Q*–*Q* plots.

All statistical analyses are performed using R 4.5.2. Linear mixed-effects models are fitted with the nlme package, and conditional logistic regression is conducted with the survival package. All tests are two-sided, with *p* < 0.05 considered statistically significant. Total physical fitness score is the prespecified primary outcome. Grade-based outcomes are secondary analyses, and raw item measurements are exploratory analyses used for interpretation rather than primary statistical inference.

### 2.7 Ethics and data governance

This study was reviewed and approved by the Ethics Committee of China University of Mining and Technology (Beijing); approval date: November 13, 2025. No separate approval number was assigned for this review. The study uses annual physical fitness assessment records collected through routine university health monitoring. All data were anonymized before analysis, and no personally identifiable information is reported. The study was conducted in accordance with the Declaration of Helsinki and relevant institutional ethical requirements.

## 3 Results

### 3.1 Baseline characteristics

The study includes 4,406 eligible students at baseline, contributing 12,795 student-year observations. Among them, 4,071 students complete all three annual physical fitness tests, corresponding to a complete 3-year follow-up rate of 92.4%. Based on baseline major in 2023, 3,284 students are classified as Engineering, 342 as Science, and 780 as HSSM. Sex distribution differs across groups: the proportion of males is highest in Engineering (77.4%) and also high in Science (71.3%), whereas females account for 59.7% of the HSSM group. Baseline grade distribution is similar across groups, with freshmen accounting for 50.4, 51.8, and 49.9%, respectively. Baseline characteristics and physical fitness indicators by discipline group are shown in Table 1.

**Table 1:** Baseline characteristics of the study cohort by discipline group in 2023.

Characteristic	Engineering	Science	HSSM
Students, *n*	3,284	342	780
Male, *n* (%)	2,542 (77.4)	244 (71.3)	314 (40.3)
Female, *n* (%)	742 (22.6)	98 (28.7)	466 (59.7)
Freshman, *n* (%)	1,656 (50.4)	177 (51.8)	389 (49.9)
Sophomore, *n* (%)	1,628 (49.6)	165 (48.2)	391 (50.1)
Total score, mean ± SD	68.62 ± 9.59	69.89 ± 9.33	70.78 ± 8.93
BMI, mean ± SD, kg/m^2^	22.65 ± 4.08	22.50 ± 4.06	21.94 ± 3.58
Pass rate, %	91.78	93.86	94.87

HSSM denotes humanities, social sciences, and management.

At baseline in 2023, total physical fitness scores are broadly similar across the three groups, with mean scores of 68.62 in Engineering, 69.89 in Science, and 70.78 in HSSM. Baseline pass rates are 91.78, 93.86, and 94.87%, respectively, and the corresponding good-or-excellent rates are 10.26, 13.74, and 15.51%. The 335 students who do not complete all three follow-up tests have lower baseline total scores than complete follow-up students, with mean scores of 64.38 and 69.49, respectively, and higher baseline BMI, with mean values of 23.94 kg/m^2^ and 22.39 kg/m^2^, respectively. Because these two groups already differ at baseline, the primary analysis retains all available eligible records, and sensitivity analyses are used to evaluate the possible influence of missing follow-up data (Figure 1).

**Figure 1:**
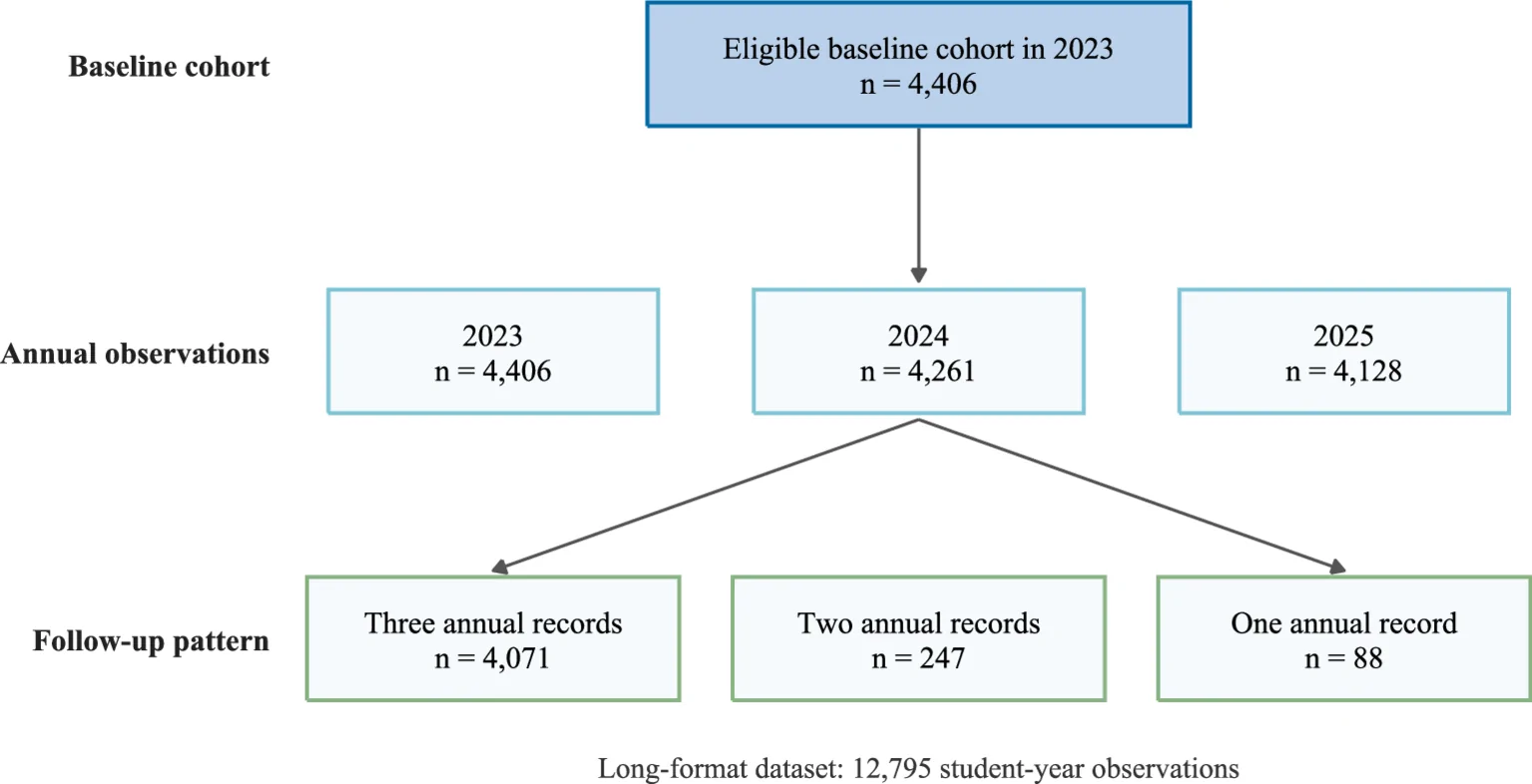
Study design and follow-up.

### 3.2 Longitudinal changes in total physical fitness score

After individual matching and formatting of the 3-year test records, linear mixed-effects models are fitted using 12,795 available student-year observations from 4,406 students. The results show a significant change in total physical fitness score from 2023 to 2025, with a year effect of *F*(2, 8,379) = 1524.06, *p* < 0.0001. The fixed-effect tests for discipline group, baseline grade, and sex are *F*(2, 30) = 3.42, *p* = 0.0458; *F*(1, 4,371) = 17.62, *p* < 0.0001; and *F* (1, 4,371) = 301.45, *p* < 0.0001, respectively. Because these variables are also modeled with interactions with year, their main effects mainly describe baseline differences among student groups, whereas longitudinal findings are interpreted through the corresponding interaction terms. The main fixed-effect tests are presented in Table 2.

**Table 2:** Fixed-effect tests from the recommended linear mixed-effects model.

Effect	Numerator df	Denominator df	*F* value	*p* value
Year	2	8,379	1,524.06	<0.0001
Discipline group	2	30	3.42	0.0458
Baseline grade	1	4,371	17.62	<0.0001
Sex	1	4,371	301.45	<0.0001
Year × discipline group	4	8,379	9.43	<0.0001
Year × baseline grade	2	8,379	732.12	<0.0001
Year × sex	2	8,379	24.13	<0.0001

The model included a random intercept for major, student-specific random intercept and time slope, and an AR(1) within-student residual correlation structure.

The year × discipline-group interaction is statistically significant, *F*(4, 8,379) = 9.43, *p* < 0.0001, indicating that the total physical fitness score does not change by the same magnitude across the three discipline groups and instead follows distinct longitudinal trajectories. The year × baseline-grade interaction is also significant, *F*(2, 8,379) = 732.12, *p* < 0.0001, showing that 3-year changes differ by baseline grade. The year × sex interaction is *F*(2, 8,379) = 24.13, *p* < 0.0001, indicating that overall changes also differ between male and female students. Stratified findings by baseline grade and sex are presented in the following section.

Model-adjusted means show declining total physical fitness scores in all three groups. In Engineering, the score decreases from 69.07 in 2023 to 63.12 in 2025, a change of −5.96 points (95% CI, −6.26 to −5.66). In Science, the score decreases from 70.53 to 62.66, a change of −7.87 points (95% CI, −8.77 to −6.97). In HSSM, the score decreases from 69.05 to 62.40, a change of −6.64 points (95% CI, −7.27 to −6.02). The largest decline is observed in Science, followed by HSSM, with Engineering showing the smallest decline. Year-by-year comparisons show that scores decrease by 0.94–2.57 points from 2023 to 2024, and by 5.02–5.56 points from 2024 to 2025, indicating that most of the overall decline is concentrated in 2024–2025. Adjusted means and changes by discipline group are shown in Table 3, and longitudinal trajectories are shown in Figure 2.

**Table 3:** Model-adjusted mean total physical fitness scores and changes by discipline group.

Year or contrast	Engineering, mean (95% CI)	Science, mean (95% CI)	HSSM, mean (95% CI)
2023	69.07 (68.51–69.64)	70.53 (68.69–72.38)	69.05 (67.95–70.14)
2024	68.14 (67.57–68.71)	67.97 (66.11–69.82)	67.96 (66.86–69.06)
2025	63.12 (62.54–63.69)	62.66 (60.79–64.54)	62.40 (61.28–63.52)
2025 vs. 2023	−5.96 (−6.26 to −5.66)	−7.87 (−8.77 to −6.97)	−6.64 (−7.27 to −6.02)

Values are model-adjusted means or mean changes with 95% confidence intervals. Estimates were standardized for baseline grade and sex. HSSM denotes humanities, social sciences, and management.

**Figure 2:**
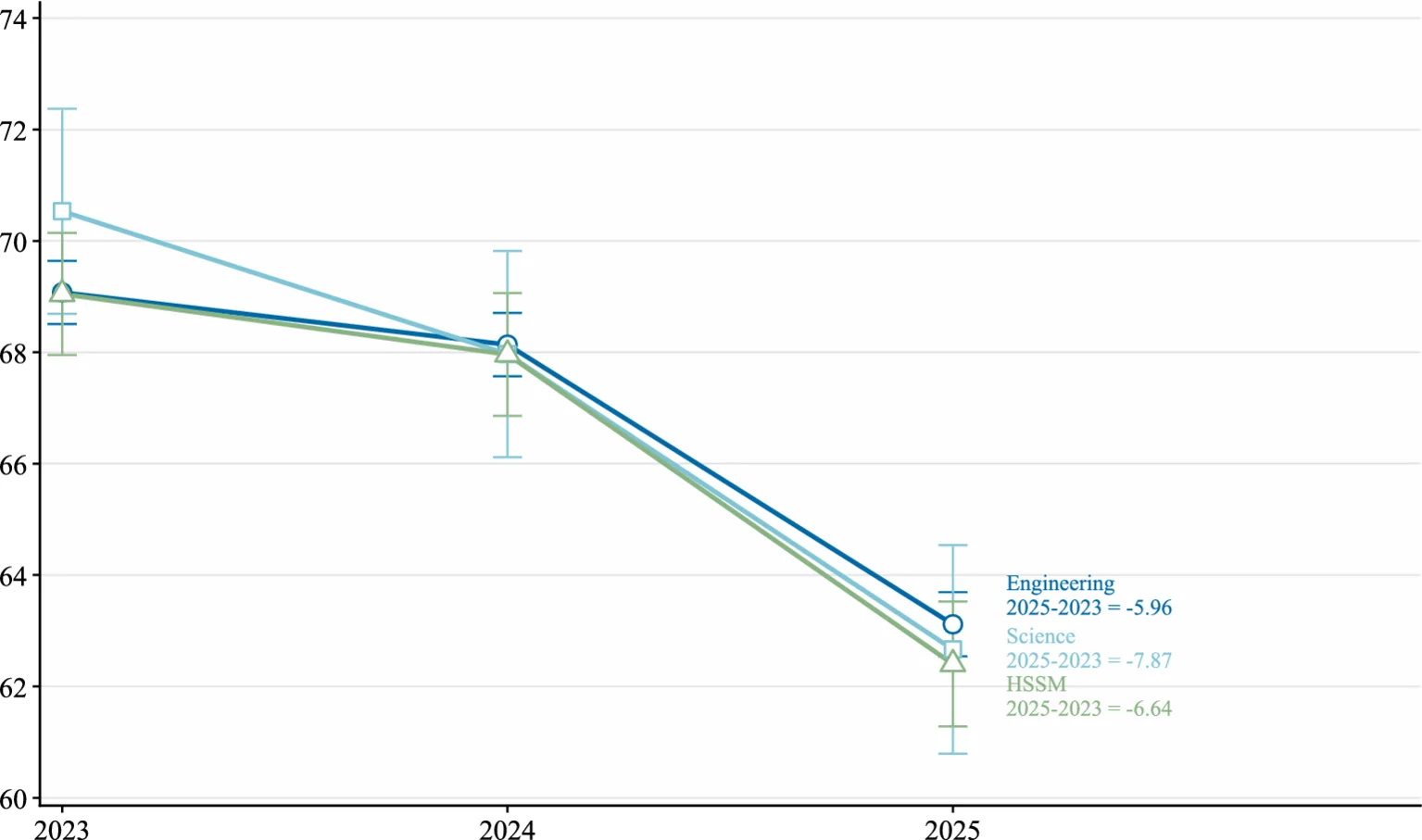
Adjusted total fitness score trajectories.

### 3.3 Modification by baseline grade and sex

Stratified results by baseline grade show that total physical fitness scores decline in both baseline-grade groups during follow-up, but the magnitude differs substantially. Among students who are freshmen in 2023, adjusted changes by 2025 are −2.74 points in Engineering, −4.65 points in Science, and −3.42 points in HSSM. Among students who are sophomores in 2023, the corresponding changes are −9.23, −11.14, and −9.92 points. The 3-year decline is clearly larger among baseline sophomores than among baseline freshmen, and Science shows the relatively larger decline in both grade strata. Adjusted changes and 95% confidence intervals by baseline grade are shown in Figure 3.

**Figure 3:**
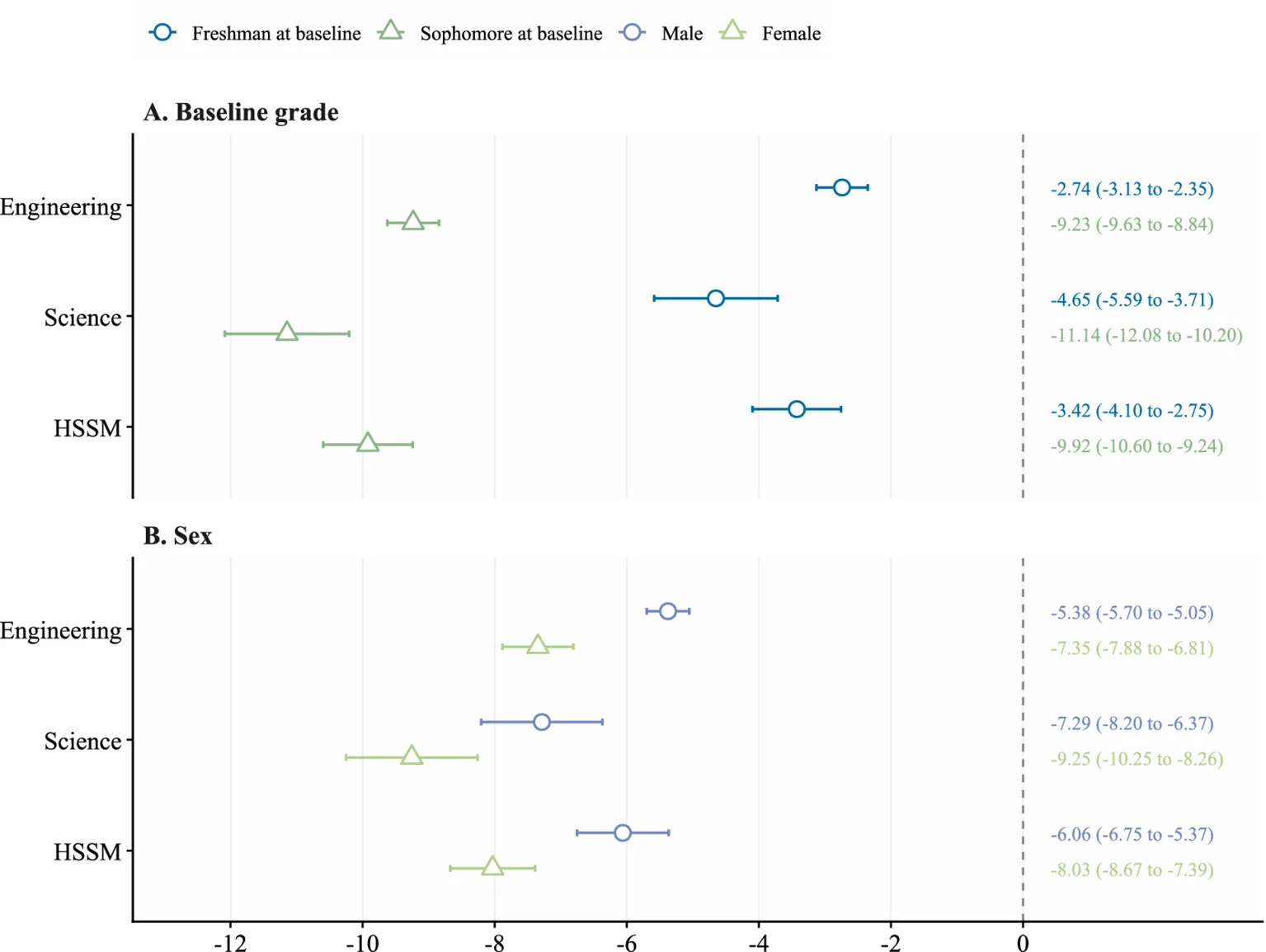
Three-year changes by baseline grade and sex.

Sex-stratified results show 3-year adjusted changes of −5.38 points in Engineering, −7.29 points in Science, and −6.06 points in HSSM among male students, and −7.35, −9.25, and −8.03 points among female students, respectively. The overall decline is larger among females. However, the year × discipline group × sex interaction is not statistically significant, *F*(4, 8,375) = 1.20, *p* = 0.3070. This indicates that there is no sufficient evidence that discipline-group differences in trajectories are clearly sex-specific, and the sex-stratified results are therefore used mainly to describe overall differences in magnitude of change.

### 3.4 Grade-based outcomes

Grade-based outcomes further show that the decline in total score is reflected in lower proportions of students meeting basic fitness standards and attaining higher fitness grades. The pass rate decreases from 91.78% in 2023 to 68.62% in 2025 in Engineering, a decrease of 23.15 percentage points. In Science, it decreases from 93.86 to 66.77%, a decrease of 27.09 percentage points. In HSSM, it decreases from 94.87 to 72.32%, a decrease of 22.55 percentage points. Pass rates decline markedly in all three groups, and grade-based outcomes with observed counts by year are shown in Table 4. In conditional logistic regression, 1,233 students whose pass status changes contribute within-student information to the model. The 500-repetition cluster bootstrap by major shows that the year × discipline-group interaction for passing is not statistically significant, *χ*^2^ = 4.33, df = 4, *p* = 0.3628, indicating insufficient evidence that the magnitude of pass-rate decline differs across the three groups. Compared with 2023, the adjusted odds ratios for passing in 2025 are 0.70 in Engineering, 0.66 in Science, and 0.67 in HSSM, suggesting that the decline in basic attainment is common across groups.

**Table 4:** Grade-based physical fitness outcomes.

Outcome	Year or contrast	Engineering	Science	HSSM
Pass, % (*n*/*N*)	2023	91.78 (3,014/3,284)	93.86 (321/342)	94.87 (740/780)
Pass, % (*n*/*N*)	2024	86.52 (2,740/3,167)	86.40 (286/331)	91.48 (698/763)
Pass, % (*n*/*N*)	2025	68.62 (2,104/3,066)	66.77 (217/325)	72.32 (533/737)
Pass-rate change, pp	2025 vs. 2023	−23.15	−27.09	−22.55
Good or excellent, % (*n*/*N*)	2023	10.26 (337/3,284)	13.74 (47/342)	15.51 (121/780)
Good or excellent, % (*n*/*N*)	2024	9.88 (313/3,167)	7.55 (25/331)	12.84 (98/763)
Good or excellent, % (*n*/*N*)	2025	3.88 (119/3,066)	1.85 (6/325)	3.26 (24/737)
Good or excellent-rate change, pp	2025 vs. 2023	−6.38	−11.9	−12.26
Excellent, % (*n*/*N*)	2023	1.19 (39/3,284)	1.46 (5/342)	0.64 (5/780)
Excellent, % (*n*/*N*)	2024	1.23 (39/3,167)	1.21 (4/331)	0.66 (5/763)
Excellent, % (*n*/*N*)	2025	0.59 (18/3,066)	0.00 (0/325)	0.41 (3/737)
Excellent-rate change, pp	2025 vs. 2023	−0.6	−1.46	−0.23

Percentages were calculated using all available observations in each year. pp denotes percentage points. Bootstrap tests for the year × discipline-group interaction were *p* = 0.3628 for passing and *p* = 0.0007 for attaining a good or excellent grade. Excellent outcomes were presented descriptively because of sparse events.

The decline in the good-or-excellent rate shows clearer between-group differences. In Engineering, the rate decreases from 10.26% in 2023 to 3.88% in 2025, a decrease of 6.38 percentage points. In Science, it decreases from 13.74 to 1.85%, a decrease of 11.90 percentage points. In HSSM, it decreases from 15.51 to 3.26%, a decrease of 12.26 percentage points. A total of 642 students experience a change in good-or-excellent status during follow-up. The bootstrap joint test shows a significant year × discipline-group interaction, *χ*^2^ = 19.24, df = 4, *p* = 0.0007. The adjusted odds ratios for attaining good or excellent status in 2025 are 0.38, 0.10, and 0.19 in Engineering, Science, and HSSM, respectively, suggesting a more pronounced loss of higher fitness grades in Science and HSSM. The corresponding odds ratios and bootstrap 95% confidence intervals are shown in Figure 4.

**Figure 4:**
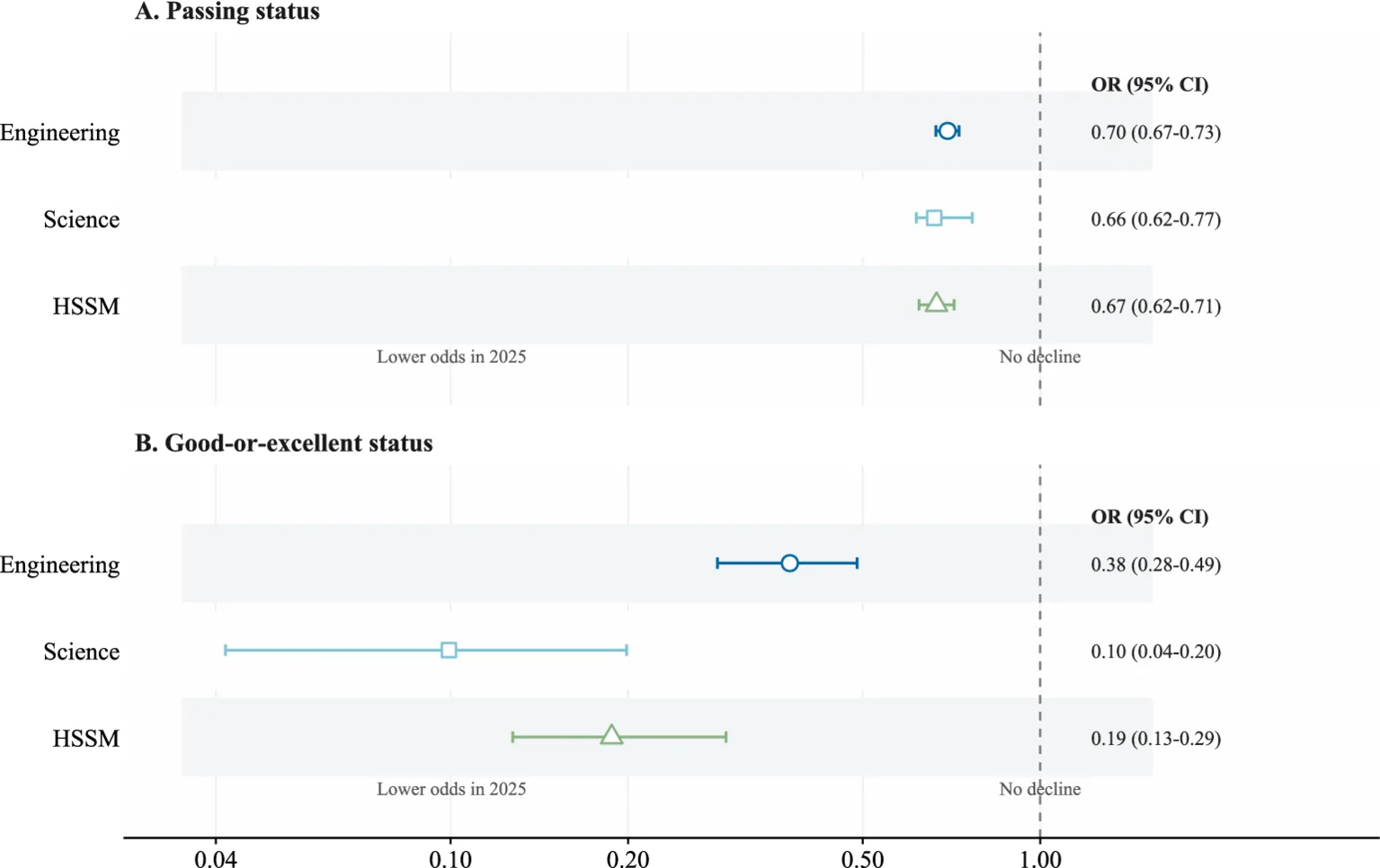
Grade-based outcome odds ratios.

The excellent rate decreases from 1.19% in 2023 to 0.59% in 2025 in Engineering, from 1.46% to 0 in Science, and from 0.64 to 0.41% in HSSM. Because excellent events are sparse and no excellent events are observed in Science in 2025, excellent status is presented only descriptively and is not used for primary statistical inference.

### 3.5 Auxiliary analysis of raw test measurements

Among students with corresponding raw item measurements, changes in specific physical fitness indicators are further analyzed to clarify the physical abilities involved in the decline in total score. The results show that not all fitness domains change in an unfavorable direction. Vital capacity and sit-and-reach generally improve across the three groups, indicating that vital-capacity measurement and flexibility are not the main domains reflected in the total-score decline. Standardized changes in raw item measurements from 2023 to 2025 are shown in Supplementary Figure S1.

In contrast, items related to speed, endurance, lower-limb explosive power, and muscular strength or endurance show more consistent unfavorable changes. The time to complete the 50-m sprint increases in all three groups, and completion times for the female 800-m run and male 1,000-m run also increase, indicating declines in speed and endurance-running performance. Standing long jump distance decreases, and the number of one-minute sit-ups among females and pull-ups among males also decreases, suggesting insufficient maintenance of lower-limb explosive power, abdominal muscular endurance, and upper-body strength. BMI increases in all three groups, but because BMI cannot distinguish fat mass from muscle mass, this result should be interpreted together with other fitness indicators.

Overall, the direction of change in raw item measurements is consistent with the decline in total physical fitness score. The decline is concentrated mainly in speed, endurance, lower-limb explosive power, and muscular strength or endurance, rather than reflecting simultaneous deterioration across all test items. Item-level analyses may be affected by item missingness, test motivation, and movement technique; therefore, they are used mainly to interpret the fitness domains involved in total-score change.

### 3.6 Robustness and sensitivity analyses

Covariance-structure comparisons show that the model including student-specific random slopes, year-specific residual heteroscedasticity, and AR(1) correlation has a lower AIC, but its variance-parameter approximate covariance matrix is not positive definite, indicating parameter-identifiability problems. The final random-slope plus AR(1) model has identifiable variance parameters and stable fixed-effect estimates. In the recommended model, the year × discipline-group interaction is *F*(4, 8,379) = 9.43, *p* < 0.0001. When the analysis is restricted to the 4,071 students with complete 3-year follow-up, the year × discipline-group interaction remains significant, *F*(4, 8,132) = 10.57, *p* < 0.0001. After removing the BMI item score from the total score, the interaction also remains significant, *F*(4, 8,372) = 12.26, *p* < 0.0001, indicating that the main finding does not depend on the BMI-score component. Inverse probability weighting for missing data gives adjusted 2023 to 2025 changes of −5.91, −7.85, and −6.68 points in Engineering, Science, and HSSM, respectively, which are closely aligned with the primary model. When the 2025 total scores of students with missing outcomes are further assumed to be 2 or 5 points lower than their conditional predicted values, the direction of decline in all three groups and the relatively larger decline in Science remain materially unchanged.

In the leave-one-major-out analysis, each of the 33 majors is removed in turn and the model is refitted. All models converge successfully, and the year × discipline-group interaction remains significant in every analysis, with the highest *p* value being 0.00021. This indicates that the main finding is not driven by any single major. The year × discipline-group interaction also remains significant after winsorizing total physical fitness scores at the 0.5th and 99.5th percentiles and after excluding observations with absolute standardized residuals greater than 4. Overall, sample restrictions, missing-data assumptions, and outlier handling do not change the main conclusions. Detailed model comparisons, missing-data analyses, and sensitivity analyses are provided in the Supplementary material.

## 4 Discussion

Using three consecutive years of physical fitness test records from 4,406 university students, this study shows a marked decline in total physical fitness scores from 2023 to 2025. This decline is not uniform. It varies by discipline group, baseline grade, and sex, and is most pronounced between 2024 and 2025. Grade-based outcomes further indicate that the problem is not limited to an increase in students failing to meet the minimum standard, but also includes a substantial loss of students who initially maintain good-or-excellent fitness. Sensitivity analyses based on missing-data weighting, complete-case samples, leave-one-major-out models, and outlier handling support the stability of these findings.

### 4.1 Overall decline in physical fitness among university students

Total physical fitness scores decline across all three discipline groups over the 3-year period, indicating that physical fitness decline during university is not confined to a single type of major (8, 24–27). The year-by-year pattern is particularly important. Scores decreased by 0.94–2.57 points from 2023 to 2024, but by 5.02–5.56 points from 2024 to 2025, suggesting that most of the decline occurred in the latter follow-up year. This pattern implies that physical fitness loss may accumulate as students move deeper into university life, rather than appearing only as a short-term fluctuation after entry. Although longitudinal studies during the COVID-19 period have also reported marked changes in university students’ physical fitness across different stages (28–30), the present findings are more likely to reflect the continuing adjustment of activity patterns, study routines, and exercise habits in a regular university setting.

The larger decrease between 2024 and 2025 may have several explanations. It may reflect cumulative reduction in structured physical education exposure, greater academic and internship pressure as students move further into their programs, reduced continuity of self-directed exercise, or changes in test motivation after repeated annual assessments. Because these factors are not directly measured, this pattern should be interpreted as a signal for further investigation rather than as evidence for a single mechanism.

A plausible explanation is that university life gradually shifts students from structured movement opportunities to more self-directed activity. In the early stage of university, physical education courses, orientation activities, and collective arrangements may still provide regular opportunities for movement. As students progress through university, specialized courses, laboratory tasks, examination preparation, internships, and postgraduate entrance pressure may increase sedentary study time and reduce the continuity of exercise participation (15, 31, 32). At the same time, physical activity during university becomes more self-directed, but autonomy does not necessarily translate into sustained participation (5, 13, 33, 34). When course requirements and organized exercise opportunities decrease, students without stable exercise habits may be more likely to experience lower exercise frequency and longer sedentary time (4, 35). This interpretation is consistent with the WHO guidelines, which emphasize that prolonged sedentary behavior is harmful to health and that sedentary time should be replaced by physical activity of different intensities (1). It is also consistent with global evidence showing that insufficient physical activity remains a widespread public health problem (2).

The decline observed in this study is therefore best interpreted as a pattern consistent with changes in activity exposure, sedentary study routines, and exercise continuity during university, rather than as the effect of any single confirmed factor. In particular, concurrent declines in endurance, speed, muscular strength, and lower-limb explosive power indicate that students may not receive sufficient regular aerobic and strength-related exercise stimuli to maintain these capacities. However, this study does not directly measure course participation, extracurricular exercise, exercise frequency, sedentary time, screen use, sleep, diet, academic stress, or sporting history. Causal explanations should therefore remain cautious. From a management perspective, these findings support greater emphasis on maintaining regular physical activity, reducing prolonged sedentary time, and supporting fitness maintenance throughout the academic year.

### 4.2 Differences by baseline grade and sex

Baseline grade is an important factor related to physical fitness trajectories (26, 36). Students who are freshmen at baseline show a 3-year decline of approximately 2.74–4.65 points, whereas baseline sophomores decline by approximately 9.23–11.14 points. This difference suggests that physical fitness maintenance may become more difficult in later stages of university study, when academic tasks, professional training, internship preparation, and postgraduate entrance pressure often become more intensive. The finding also indicates that university health promotion should not be concentrated only at entry, but should extend across the whole university period, especially after compulsory or structured physical activity opportunities decrease. However, baseline grade contains both academic-stage and cohort information. Therefore, the larger decline among baseline sophomores should be interpreted as evidence of stage-related vulnerability during university, rather than as proof that grade level itself directly causes the decline.

Female students show larger adjusted declines than male students across the three discipline groups, whereas the year × discipline group × sex interaction is not statistically significant. This suggests that sex is related to the overall magnitude of physical fitness change, but does not materially alter the discipline-specific trajectory pattern. Because male and female students complete different strength and endurance items, and because total scores are calculated using sex-specific standards, the sex-related results should be interpreted as group-level change patterns rather than direct comparisons of identical test performance. Previous studies among university students have also reported sex differences in physical activity, exercise participation, and lifestyle behaviors (13, 33, 37, 38).

### 4.3 Discipline-specific trajectories and possible explanations

All three discipline groups show declining total physical fitness scores, but the magnitude of decline differs (14, 15, 36). From 2023 to 2025, adjusted scores decrease by 5.96 points in Engineering, 7.87 points in Science, and 6.64 points in HSSM, with the largest decline observed in Science. The significant interaction between year and discipline group indicates that these differences reflect distinct longitudinal trajectories rather than simple baseline differences. Leave-one-major-out analyses further show that the interaction remains significant after removing each major in turn, suggesting that the discipline-group difference is not driven by a single major.

Curriculum-plan data provide a useful context for interpreting these trajectories. Based on major-level data from the undergraduate teaching quality reports and weighted by the baseline sample size in this study, practical-teaching credits account for approximately 35.48, 31.64, and 31.08% of credits in Engineering, Science, and HSSM in the 2022–2023 academic year, and 30.08, 26.18, and 24.02% in the 2023–2024 academic year. Across both academic years, Engineering has the highest proportion of practical teaching, which is directionally consistent with its relatively smaller decline in total physical fitness score. This does not mean that practical teaching is equivalent to physical activity, but it suggests that differences in training structure and daily academic routines may be relevant when interpreting discipline-specific fitness trajectories.

This interpretation should remain cautious. Practical-teaching credits do not directly measure exercise intensity, sedentary time, extracurricular physical activity, or sporting history. Laboratory work, internships, and engineering training may involve more standing, walking, or campus movement, but they may also include static tasks. Curriculum-plan data should therefore be understood as contextual indicators of the academic training environment rather than direct behavioral measurements. The Science group in this study consists of mathematics and applied mathematics and geophysics. The consistency of leave-one-major-out and missing-data analyses supports the stability of the observed trajectory within the present sample, but multi-university studies are needed to examine whether similar patterns appear across broader science programs.

### 4.4 Changes in grade-based outcomes and their management implications

Pass rates decline markedly in all three groups, and the interaction between year and discipline group is not statistically significant. This indicates that the decline in basic passing status is a shared problem across the university, rather than a pattern confined to a single discipline group (1, 4, 5). Remedial action targeting only a small number of failing students is therefore unlikely to change the overall trend. Universities need to provide basic exercise support across grades, including continuity in physical education, protected time for in-class and extracurricular activity, and curricular arrangements that reduce the long-term displacement of regular exercise by academic demands (39–42).

The grade-based results also reveal a problem that is easily overlooked. Physical fitness change during university is not only reflected in the transition from passing to failing, but also in the movement of students from good-or-excellent status to lower categories. The good-or-excellent rate decreases by 11.90 percentage points in Science and 12.26 percentage points in HSSM, compared with 6.38 percentage points in Engineering. The cluster bootstrap by major further supports discipline-group differences in good-or-excellent outcomes. These findings suggest that university physical fitness management should not focus only on whether students reach the 60-point threshold, but should also consider whether students can maintain a higher level of physical fitness over time.

Excellent rates are low in all groups, and no excellent results are observed in Science in 2025. Because excellent events are limited, stable inference about between-group differences is not possible. Nevertheless, this pattern reinforces the concern that using the passing threshold as the main management goal may be insufficient for improving overall physical fitness. Universities can use continuous annual testing to identify students whose total scores or fitness grades are declining and provide exercise guidance and staged interventions before they fall below the relevant grade threshold (43, 44).

The magnitude of the score change also has practical relevance. A decline of approximately 6–8 points is large enough to move many students across grade thresholds, which is reflected in the marked reduction in pass rates and good-or-excellent rates. Therefore, the findings should not be viewed only as statistically significant model estimates; they represent changes that can alter students’ classification within the university physical fitness management system.

### 4.5 Insights from raw test measurements

Compared with item scores, raw measurements directly reflect changes in running time, jump distance, and repetitions completed, reducing the influence of scoring standards and grade thresholds on interpretation. The item-level results show that the decline in physical fitness is not a simultaneous deterioration across all domains. Vital capacity and sit-and-reach improve, whereas the 50-m sprint, endurance running, standing long jump, sit-ups, and pull-ups show unfavorable changes (9, 10). This pattern suggests that the decline in total score is mainly related to speed, endurance, lower-limb explosive power, and muscular strength or endurance, rather than a uniform decline in all measured capacities.

These item-specific changes are physiologically plausible. Speed, endurance, jumping ability, and muscular fitness usually require regular aerobic activity, strength-related training, and sufficient exercise intensity to be maintained (45–48). Sprinting and jumping performance are also closely related to lower-limb strength and mechanical characteristics (20–22, 49). Similar domain-specific declines have been reported in surveillance studies when structured physical activity and daily movement opportunities are restricted, with larger changes observed in cardiorespiratory fitness and muscular fitness (50). Although those studies differ from the present study in population and context, they support the interpretation that the domains most affected in this study are those more sensitive to reduced or inconsistent exercise exposure.

BMI increases in all three groups, but BMI should be interpreted cautiously because it cannot distinguish fat mass from muscle mass and does not independently represent overall physical fitness (7, 51–56). After removing the BMI item score from the total score, the interaction between year and discipline group remains significant, indicating that discipline-specific trajectories are not fully explained by changes in the BMI-score component. Item-level analyses may also be affected by missing values, test motivation, and movement technique. Therefore, these analyses should be treated as explanatory evidence for the direction of physical fitness change, rather than as proof that insufficient exercise or weight-status change directly causes the decline in total score.

From a health-management perspective, universities should retain and continuously compare raw measurements, rather than relying only on total score and grade. Sustained declines in endurance running, the 50-m sprint, standing long jump, sit-ups, or pull-ups can help identify whether a student needs more aerobic exercise, speed training, or strength training. Regular monitoring of these indicators may support earlier identification of weak areas and more precise stratified intervention (1, 39, 40, 45, 46, 57, 58). Because item tests may still be influenced by test motivation, movement technique, and testing conditions, their changes should be interpreted using repeated measurements under standardized testing procedures.

### 4.6 Implications for university physical health promotion

The findings indicate that university physical fitness management should shift from annual test evaluation toward continuous health promotion throughout university. Annual testing can identify problems, but it cannot replace daily physical activity. Universities first need to establish basic exercise support for all students (1, 4, 5, 42–44, 59), with particular attention to fitness maintenance after compulsory physical education courses decrease. Fixed activity periods, extracurricular training courses, and short high-frequency activity sessions may be feasible approaches, but their effects require evaluation in future intervention studies.

Interventions should also consider students at different stages and different risk levels. The clear decline in all three groups indicates the need for a basic intervention strategy that covers all students. The larger decline among baseline sophomores suggests that later stages of university may require more physical activity support (26, 36). The more pronounced loss of good-or-excellent status in Science and HSSM indicates that more convenient exercise opportunities may be needed in relation to each university’s course rhythm. Discipline group can help with preliminary risk identification, but it cannot replace assessment of individual physical fitness and exercise behavior, and it should not be used to label students in specific majors.

Health-promotion goals should not be limited to reducing the failure rate. Universities also need to address the continuous loss of students with good or excellent fitness and use repeated annual testing to identify students whose total scores, grades, or item performances are declining. Students may be classified into stable, mildly declining, and continuously declining groups according to the degree of change, with routine exercise guidance, targeted training, and individualized intervention provided before students fall below relevant standards. Training content should correspond to the fitness domains that actually decline. This study identifies endurance, speed, lower-limb explosive power, and muscular strength as key capacities requiring maintenance. Universities can increase aerobic endurance training, basic strength training, and speed training within existing physical education courses and extracurricular activities (1, 39, 40, 42–46, 49), and regularly monitor changes in endurance running, standing long jump, and strength items. For students with sustained increases in BMI, body composition, diet, and physical activity should be considered together, rather than making judgments based on BMI alone.

Finally, universities should establish a mechanism for continuous use of physical fitness data. Total scores, grades, and raw item values from different years should be linked at the individual level to form student-specific fitness trajectories (14, 24, 25, 27). Intervention effects should be evaluated through subsequent measurements, while physical education participation, extracurricular exercise, sedentary time, and training completion should also be recorded. Transforming annual testing into a continuous monitoring and feedback tool can help identify which students, stages, and fitness domains are involved in physical fitness decline and can guide adjustment of physical education and health-promotion strategies.

## 5 Strengths and limitations

This study has several strengths. First, it uses individually linked annual physical fitness records across three consecutive years, which allows direct examination of within-student change rather than relying only on repeated cross-sectional comparisons. Second, the primary model accounts for student-level repeated measurements, individual variation in change, major-level clustering, and within-student residual correlation. Third, the analysis integrates total scores, grade-based outcomes, and raw item measurements, which helps connect statistical change with practical implications for university health management. Finally, multiple sensitivity analyses, including complete follow-up analysis, inverse probability weighting, missing-outcome stress testing, leave-one-major-out analysis, and outlier handling, support the robustness of the main findings.

This study also has limitations. It is based on one university, and the discipline-group structure reflects the majors represented in this institution. Although the sample is large and includes 33 majors, external generalization to other universities should be made with attention to differences in curriculum structure, student composition, and campus physical activity environments. The Science group includes mathematics and applied mathematics and geophysics; therefore, discipline-specific results should be interpreted within this institutional composition. The study does not directly measure sporting history, physical activity frequency, sedentary time, screen use, sleep, diet, academic stress, course participation, or physical education attendance. Therefore, behavioral and curriculum-related explanations should be interpreted as plausible interpretations rather than causal proof. Raw item analyses may also be affected by missing item records, test motivation, and movement technique, although the test month, venue, equipment, scoring standards, and organization procedures remain consistent across years. Finally, the observational design cannot establish causal effects of discipline group or curriculum characteristics on physical fitness decline.

## 6 Conclusion

In this 3-year longitudinal study of university students, total physical fitness score declines substantially from 2023 to 2025, with most of the decline concentrated in 2024–2025. The decline is observed across Engineering, Science, and HSSM students, but its magnitude differs by discipline group, baseline grade, and sex. Science shows the largest decline in total score, while Science and HSSM show greater loss of good-or-excellent fitness status. Raw measurement analyses indicate that the decline is concentrated mainly in speed, endurance, lower-limb explosive power, and muscular strength or endurance, rather than reflecting deterioration across all test domains. These findings support a shift in university physical fitness management from annual outcome evaluation toward continuous monitoring, early warning, and stratified intervention across the university period, while acknowledging that behavioral mechanisms require direct measurement in future studies.

## Data Availability

The data analyzed in this study is subject to the following licenses/restrictions: The dataset contains institutional records from three years of student physical fitness monitoring and is not publicly available due to privacy and ethical restrictions. De-identified data may be made available by the corresponding author upon reasonable request, subject to approval by the relevant institution and ethics committee. Requests to access these datasets should be directed to Yang Sanjun, sanjun_yang@163.com.
